# Pathology and Treatment of Dysentery

**Published:** 1852-09

**Authors:** 


### EDITORIAL DEPARTMENT.

---

*Pathology and Treatment of Dysentery.* — Our readers will find in the original department of this No., a communication from an esteemed correspondent, Dr. Hunt, which we insert with pleasure, notwithstanding we are obliged to dissent from the theoretical and practical views therein contained. Free discussion, opposing arguments, and facts pertaining to both sides of mooted questions, contribute to the development of truth. It is one of the most important of the functions of a medical journal to afford facilities for these objects. We are always glad to receive articles designed to controvert opinions advanced either by our correspondents, or ourselves, if written in that spirit of candor and courtesy which is an essential element of a philosophical temper.

Dr. Hunt attributes the cases of epidemic dysentery occurring in his neighborhood to malarial causes. The facts which he adduces, however, appear to us only sufficient for a bare presumption on this point. The malaria to which he attributes the origin of the disease, is that supposed to emanate from vegetable matter in a state of decomposition. It has been customary to attribute morbific emanations to this source for a long period, but it is questionable whether the doctrine has much to sustain it beyond tradition. There is abundant evidence to prove that the special cause of intermittent and remittent fevers, commonly known as marsh miasmata, is, to say the least, not uniformly produced by vegetable decomposition. Whether it is ever due to the latter alone, admits of doubt. But not to pursue this topic, the supposition is made by Dr. H. as bearing on the hepatic pathology of dysentery. He apparently takes it for granted that if the disease be malarial in its origin, the liver is implicated as a matter of course. This, too, is an idea which has the sanction of tradition; but, for one, we must withhold assent until it is shown to rest on facts, instead of mere theoretical assumption. The term *bilious* is often enough used by patients and physicians to create for it some significance, if there be any virtue in the aphorism *vox populi est vox Dei.* This criterion of truth, however, if it be applicable to politics, is certainly not to medical science. For us, the term is devoid of any special meaning. It does not express any well defined pathological condition. It has only a loose, conventional, popular signification, which would make it convenient sometimes in order to satisfy the very natural desire of patients that their disorders should have some name. With our notions, we cannot ever say to a patient "Sir, you are bilious." Our correspondent with his views can doubtless consistently speak thus; but for us it would be more than an absurdity. Dr. H. is by no means alone in the use of the term; but we should be glad to have some one of the many who daily employ it, reduce to writing a rigorous definition of the sense in which they understand it. Do they mean too much or too little bile; or bad bile; or obstructed bile?

A term used to denote a morbid condition must have reference to something tangible or appreciable. Now what is the ensemble of symptoms which characterises the bilious state? When this question is answered, we shall be ready to ask upon what grounds the symptoms mentioned are assumed to be derived from the pathological change which is assumed to exist. The truth is, as it seems to us, the indefinite notions so generally entertained on this subject are entirely gratuitous, and the sooner they are discarded, the more favorable is the attitude for real progress in pathological knowledge.

Our correspondent mentions certain precursors of an attack of dysentery, which he appears to think favors the supposition that this disease is preceded by "hepatic derangement," to use an expression often employed by physicians as a synonym of the more popular phrase "*bilious disorder*." So far as our impressions go, the cases of dysentery that have fallen under our own observation, have not usually presented the premonitory symptoms mentioned by Dr. H. We have found the attack generally to be ushered in by simple diarrhœa of greater or less duration, or occurring abruptly, i. e. the dysenteric symptoms exhibited at the outset. It is true we have not looked for precursory symptoms referable to the liver; that is to say, we have not judged of the prodromes by this pathological preconception. But we are not prepared to admit that, on this account, our impressions are less likely to be correct.

Our correspondent states that dysentery, in the cases observed by him, has not consisted of pure inflammation. If he means by this that the pathology of the disease involves something ulterior to the local affection, we are not prepared to take issue with him on that point. This is very far from an admission that the ulterior morbid condition is hepatic congestion. Here we may ask for positive proof that congestion of the liver obtains in dysentery. Assuming, for the sake of argument, that this condition does exist, we may then ask what proof is there that the dysenteric affection is dependent on the hepatic congestion. Not only is there no positive evidence of such a dependence, but facts lead to a different conclusion. We know that congestion of the liver does exist under certain circumstances; for example in the lesion of that organ called cirrhosis. The *portal circle* then without doubt becomes engorged, and what is the consequence? Does dysentery supervene? Are the symptoms of dysentery embraced among the legitimate results of cirrhosis? Not at all, even when the portal vessels are distended sufficiently to occasion a mechanical expression of more or less of their serous contents through the coats of the vessels into the peritoneal cavity. Ascites ensues, but not dysentery. Dysentery probably involves an occult proximate cause inducing the intestinal affection; but this is true of other local inflammations frequently, if not generally. The disease is local in the same sense hat typhoid pneumonia is an inflammation of the lungs. It is a disease characterized by inflammation of the mucous membrane of the large intestine, but involving something more than, and prior to this, especially in its epidemic form; but in what this prime essential pathological condition consists, in the existing state of the science, we cannot say. It is easier, it must be confessed, to show that it is not hepatic congestion, than to substitute any other explanation, which, with our present knowledge, would, of necessity, be equally hypothetical. We do not understand Dr. H. to deny the existence of inflammation in all cases of dysentery. Had he examined *post mortem* in his fatal cases, he would have found abundant evidences thereof. From the tenor of some articles that have occasionally fallen under our notice, we have been led to think that the fact of local inflammation being present in this disease, is not sufficiently appreciated by all practitioners. If so, it doubtless arises from examinations after death not having been made or witnessed. The morbid anatomy of dysentery should be well understood, with reference not only to the pathology, but the treatment of the disease. In this connection it may not be amiss to quote the results deduced from 231 dissections made in the Prague Hospital, by Dr. Finger, for which we are indebted to the British and Foreign Med. Chir. Review, No. for July, 1852.

This large number of dissections were made by Dr. Finger between the years 1846 and 1848, during which period dysentery prevailed, more or less, as an epidemic, at Prague. We give the following abstract by the reviewer, which we think will interest the reader:

1. The first or elementary form is termed intestinal catarrh, with especial implication of the follicles. In this stage, the large intestine viewed from without appeared more or less reddened, and was moderately distended with air; if the disease had been of short duration, its coats did not appear thickened. When the intestine was opened, a yellow, brownish, grayish red, or sometimes purulent-looking fluid, more or less intermixed with fæcal matter, flowed out. The mucous membrane was reddened in patches of variable size, and was usually covered with a grayish-white or grayish-red pulpy substance; underneath this the membrane itself was softened, and was easily removed in the form of a grayish-red jelly-like pulp. The solitary glands were swollen to the size of millet or hemp seeds (*zu Hirse bis Haufkorngrose*), by distension with transparent jelly-like mucus, which could be forced out by pressure; and were often surrounded by vascular zones. Afterward the mucus became opaque, thick and purulent, and then arose like (hempseed or lentil-sized) ulceration of the glands; these were at first solitary, then ran together by the destruction of the intervening mucous membrane, and formed ulcers varying in size from the original ulcer to that of a sixpence and larger; the borders of the ulcers were loose, ragged, swollen, and usually undermined. By the farther extension of these ulcers, very frequently large ulcerations arose, which spread over great part of the colon, and were connected by bridge-like portions of mucous membrane. Here and there little pits were formed by suppurated follicles, which sometimes passed through and even perforated the peritoneum. The submucous tissue formed, however, the most frequent floor of the ulcers.

When the process extended into the small intestines, it was found in an earlier stage than in the large, showing the affection of the ilium was subsequent to that of the colon. The follicles were here also affected. In two cases of the 231, the jejunum and ilium were diseased, while the large intestines were healthy. Of the large intestines, the transverse colon and the sigmoid flexure were most affected. In some cases there was extensive catarrhal redness (*catarrhalischer Rothung*) of the small and large intestines, with numerous small, isolated, usually round aphthous erosions.

The tendency to gangrene of the intestines was not so great in the cases of pure follicular disease, as when the next form was combined with it; and when it did occur, it attacked chiefly the undermined mucous membrane. By longer duration of the follicular disease, which sometimes lasted even for many months without attendant croupous exudation on the mucous membrane (which was chiefly the case in children and feeble anæmic adults), the intestinal coats were thickened and anæmic, and the mucous and submucous coats were softened, infiltrated with serum, and thereby thickened, either pale as if washed, or of dark, smutty, or slate-grey color from pigment, or they presented yellow or gray projections (*Buckeln*), between which ran enlarged and varicose vessels. The follicles were often collapsed, with sometimes a brown or black circle of pigment. The process could, however, be seen in various stages; in some parts the follicles contained fresh infiltration-matter, or had suppurated; in other parts they had healed. By successive attacks of portions of the mucous membrane, the disease thus lasted sometimes for months.

The cicatrization occurred by the borders of the ulcers becoming covered with a white more or less thick callus, which joined on to a more or less shining smooth tissue, which spread over the base of the ulcer. The cicatrices sometimes formed hard fibroid intersecting cords and elevations, by the side of which were often still swollen and suppurated follicles. The submucous tissue was, like the mucous, changed into a firm, thick, fibroid formation.

The mesenteric glands were found unchanged in cases of short duration, they subsequently became swollen, injected, succulent, and softened, but without evident infiltration.

2. The foregoing changes were present in every case but one; but in addition there existed in many cases what Finger calls a "croupous exudation," which was either spread uniformly over great part of the mucous membrane, or was collected near the folds of the colonic mucous membrane. In color it was whitish, grayish-white, or reddish; it had a granular (*korniges*) or scaly appearance, and was usually easily detached. Under the microscope, besides epithelium, and mucous corpuscules, there was an amorphous molecular mass, often mixed with blood-discs, or at a later date with pus-cells. According to the varying condition of this exudation, the intestinal contents presented various characters, and sometimes, by the suppuration of the exudation, were chiefly made up of pus mixed with blood. Underneath the exudation the mucous membrane was often softened, sometimes to a great extent, was at first reddened, and afterward in chronic cases became anæmic, presented pigment-discoloration, and was thickened from serosity. The follicular lesion was combined with this exudation in 232 cases, and was absent in one, and was generally far advanced before the croupous exudation commenced on the other portions of the mucous membrane. Sometimes the follicular ulcers were themselves covered with croupous exudations, which contained no pus cells. Many stages were generally seen in the same intestine; in some places the mucous membrane, deprived of epithelium and covered with exudation, presented many ecchymosed vessels running through it, and was easily detached in the form of a reddish pulp; at other places the membrane was bluish or slate colored, elevated in knobs, and with its submucous tissue converted into a thick callus-tissue, while the follicles of the neighboring membrane were filled with purulent infiltration, or were destroyed by ulceration.

The tendency to gangrene was considerable when there was croupous exudation, but was much less marked in the follicular disease. It occurred in about one-third of the whole number of cases. It was confined to small follicular ulcers in only three or four cases; in other instances it spread over a considerable extent of surface. The gangrene often occurred in a very short time, especially in persons previously healthy; and it was observed with surprise, that it was less marked in persons who, before the attack of dysentery, had labored under a blood-disease, such as puerperal fever, typhus, gangrenous tuberculosis or cancer. The coincident or subsequent conditions of the intestines were, peritonitis without perforation, peritonitis with perforation and effusion, and narrowing of the intestine from healing of the ulcers.

The reliance on calomel in the treatment of dysentery is predicated, theoretically, on the supposed causative connection of the latter with an affection of the liver. They who disclaim the hepatic hypothesis, of course must reject the inference which leads to the mercurial treatment. We are not prepared to deny in toto that calomel exerts any curative influence in dysentery; but we see no rational grounds for its salutary action in this disease aside from those which apply generally to the treatment of inflammations. The value of this remedy in the disease under consideration must be determined mainly by the results of experience. Dr. H. gives a single case in which mercury being continued while all the symptoms were of a very unpromising character, the patient recovered. The reflection which the case suggested was natural, but he will hardly claim that its correctness is proved. He states that in some instances in which the liver did not seem to be implicated, a full opiate appeared to cut short the disease. But if the liver be implicated in a portion only of cases of the disease, it certainly does not furnish an essential element of its pathology. To be an essential element the hepatic congestion must be constant; otherwise it is only an incidental or accidental event. It must be considered rather inconsistent with the hepatic doctrine that a remedy supposed to lock up the biliary and other secretions, like opium, should be competent, in any cases, to arrest at once the disease. Not to dilate on the subject of treatment, for which we have not, at present, either time or space, we will reiterate our conviction that mercury in the treatment of dysentery has been vastly overrated. This conviction is based on comparative observations of the treatment of cases with, and without this remedy; and we venture to say that if our correspondent will employ opiates, without the addition of calomel, in a series of cases, his own observations will lead him to the same conclusion.

In estimating the effects of remedies in this disease, it is to be borne in mind that in its ordinary sporadic form it is very rarely fatal. Cases will recover pretty uniformly under different, and opposite plans of management. It is also to be considered that in its graver epidemic forms the intestine is apt to suffer lesions, which, in a certain proportion of cases, render recovery almost impossible. The relative proportion of cases may not, therefore, be a reliable test of the efficacy of management. We will add that our observations have led us to regard opium as emphatically the efficient remedy in the treatment of epidemic dysentery. The efficacy of opium in the graver forms of the disease, depends on its administration with reference not to quantity, but effect. There are certain conditions of disease in which the narcotic power of opium appears to be antagonized in a remarkable degree. This is true of some cases of dysentery. It may be given largely without any evidences of narcotism, and *must* be given largely to ensure efficient service. On this subject we may report some facts hereafter. Our desultory remarks are already so extended that we must bring this article somewhat abruptly to a close. We had no intention, when we commenced writing, of occupying more than a few lines. The subjects, however, are of so important a character, that it would be far easier to continue writing, than to find a convenient stopping place, were the article much longer than it is.

We are much obliged to Dr. H. for this, as well as former contributions to our pages; and should he do us the favor to practice upon the suggestion we have thrown out, we hope he will give us the results. If he resolves to give the opiate treatment a fair trial we beg him to bear in mind the principle to give the remedy with a view to effect; that is, in grave cases to prescribe doses which will be borne without narcotism, however large. If he does this he will be surprised at the amount which may be given with safety and advantage.

